# Development of Accelerated Coronary Atherosclerosis Model Using Low Density Lipoprotein Receptor Knock-Out Swine with Balloon Injury

**DOI:** 10.1371/journal.pone.0163055

**Published:** 2016-09-15

**Authors:** Manabu Ogita, Katsumi Miyauchi, Akira Onishi, Shuta Tsuboi, Hideki Wada, Hirokazu Konishi, Ryo Naito, Tomotaka Dohi, Takatoshi Kasai, Yuko Kojima, Robert S. Schwartz, Hiroyuki Daida

**Affiliations:** 1 Department of Cardiovascular Medicine, Juntendo University Graduate School of Medicine, Tokyo, Japan; 2 Transgenic Animal Research Center, National Institute of Agrobiological Sciences, Tsukuba, Japan; 3 Division of Biomedical Imaging Research, Biomedical Research Center, Juntendo University Graduate School of Medicine, Tokyo, Japan; 4 Minneapolis Heart Institute and Foundation, Minneapolis, Minnesota, United States of America; Universiteit van Amsterdam, NETHERLANDS

## Abstract

**Background:**

Several animal models have facilitated the evaluation and pathological understanding of atherosclerosis, but a definitive animal model of coronary atherosclerosis is not available. We therefore developed low density lipoprotein receptor knockout (LDLR-KO) pigs with hypercholesterolemia, a model which rapidly developed coronary atherosclerosis following balloon injury.

**Methods and Results:**

We deleted LDLR exon regions from cultured porcine fetal fibroblasts and cloned LDLR knockout (LDLR-KO) embryos microinjecting fetal fibroblast nuclei into enucleated oocytes. Twelve LDLR-KO pigs were fed a 2.0% cholesterol and 20% fat diet. Baseline serum LDL cholesterol level was 510.0±86.1 mg/dL. Balloon injury was created in 46 coronary segments and necropsy were obtained 2, 4, 8 and 12 weeks later. Coronary artery sections were reviewed to evaluate lesion progression. We found lipid accumulation with foam cells and inflammatory cells beginning four weeks after balloon injury. The mean ratio of macrophages to plaque area was significantly higher in the four- weeks and eight-week animals compared with those at 2-weeks (8.79% ± 5.98% and 17.00% ± 10.38% vs. 1.14% ± 1.88%, P < 0.0001). At 12 weeks the ratio decreased toward the level at 2 week level (4.00% ± 4.56%, P = 0.66 vs. baseline). Advanced coronary atherosclerotic lesions contained lipid pools at eight-weeks with fibrous components beginning at 12 weeks.

**Conclusions:**

We developed a model of rapid coronary atherosclerosis using LDLR KO pigs with balloon injury. This model may be useful for preclinical evaluation of medication or devices, and may also help investigate mechanisms of plaque progression.

## Introduction

Coronary atherosclerosis is the leading cause of death and disability in developed countries and is a serious health problem worldwide [1]. Understanding the pathophysiological mechanisms of progression would be useful to evaluate prevention strategies. Mice and rabbits fed high-fat diets spontaneously generate atherosclerotic aortic plaque [2, 3], but large animal models that adequately mimic coronary atherosclerosis are not widely available [4, 5].

Swine coronary arteries are anatomically and physiologically similar to humans [6, 7]. Many preclinical studies use pig coronary models, and these have contributed to advances in coronary intervention [8]. However, preclinical studies have focused on non-atherosclerotic normal coronary swine arteries raising questions about applicability to humans. Thus, much effort is directed toward developing an atherosclerosis model similar to human coronary lesions. Experimental findings show that domestic swine fed high-cholesterol/high-fat diets spontaneously develop atherosclerosis [9, 10]. Rapacz found that coronary atherosclerosis develops in pigs with genetically inherited hyper LDL-cholesterolemia [4, 5]. Several swine coronary atherosclerosis models have been induced by combining diabetes, hypercholesterolemia [11], with and needle injury [12]. Widespread application of these models is limited because of the long period required for advanced atherosclerotic lesions and high cost. These consideration suggest the need for less expensive and rapidly developing-swine model. We thus created an accelerated model of coronary atherosclerosis using a low density lipoprotein receptor knock-out (LDL-R KO) pig with balloon injury and a high lipid-rich diet.

## Material and Methods

### Ethic Statement

Experiment with gene recombination were performed in agreement with the Gene Recombination Experiment Security Committees University of Juntendo University (No. DNA22-44) and the National Institute of Agrobiological Science (No. 500035). Animal Care and Use Committee of Juntendo University (No. 1036) and the National Institute of Agrobiological Science (No. H18-038) approved the entire study and the experiments were performed in accordance with the NIH guidelines (Guide for the care and use of laboratory animals). All pigs were housed and monitored with veterinary care at the Center for Biomedical Research Resources, Juntendo University. General condition of pigs was monitored directly by veterinary technicians of the house every two-hour. Standard procedures for animal husbandry were followed. All procedure was performed under anesthesia with ketamine (30 mg/kg) and xylazine (3 mg/kg) intramuscularly, and maintained with 1% - 2% isoflurane by ventilator after intubation. Continuous hemodynamic and electrocardiographic monitoring was done. The pigs were euthanized using intravenous barbiturate commercial euthanasia solution by ear vein (50 mg/kg). Body temperature and respiration rate was used as humane endpoint in the present study.

### Production of cloned LDL-R-targeted pigs

Cloned LDL-R-targeted pigs were produced as previously described [13]. In brief, the conventional targeting vector for porcine LDLR gene was constructed so that a major part of exon 4 was replaced by the neomycin resistance gene. This vector was introduced into fetal fibroblasts from Landrace x Large White crossbred pigs using a Gene Pulser II (Biorad). The targeted cell clones were screened by PCR and then used as donor cells for nuclear transfer. Nuclear and subsequent embryo transfer were performed as previously described [14]. The cloned fetuses were collected at 39 and 72 days of gestation to confirm the targeting events by southern blotting and to obtain large cell populations for further nuclear transfer. The cell populations which were confirmed for targeting were used for secondary nuclear transfer. The F1 progeny was produced by artificial insemination or in vitro fertilization (IVF) using epidyimal sperm collected from clones as previously described [15]. The F2 and later progeny including homozygously LDLR-targeted pigs were produced by conventional breeding methods using heterozygously LDLR-targeted F1 pigs.

### Animals

**Fig 1:**
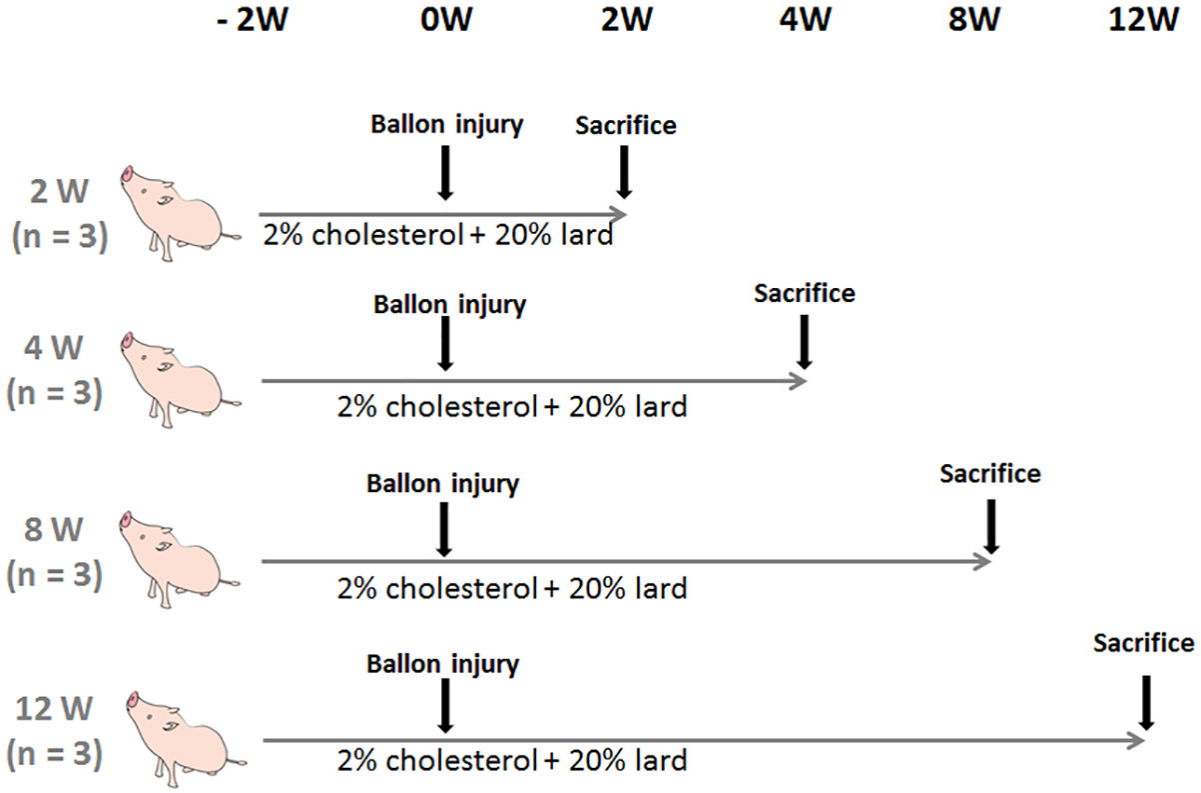
Study protocol. Twelve two-month-old LDLR-KO pigs were randomly allocated to four groups (n = 3 each) and fed with a diet containing 2.0% cholesterol/20% fat throughout the study. The coronary arteries were injured using balloons and then the pigs were sacrificed at 2, 4, 8 and 12 weeks later. Coronary plaque development was accelerated in all pigs by angioplasty of left anterior descending and left circumflex arteries.

Twelve juvenile LDLR-KO pigs (Sus scrofa, aged 2 to 3 months and weighing 20–30 kg) were randomly allocated into four groups (n = 3 each) and euthanized at 2, 4, 8, 12 weeks after balloon injury. All pigs were fed with a diet comprising of 2.0% cholesterol/20% lard throughout the study to develop and maintain hypercholesterolemia. Angioplasty was performed in the left anterior descending (LAD) and left circumflex coronary arteries (LCX) of all groups after two weeks to accelerate coronary plaque development (Fig 1). Blood samples were obtained at baseline, immediately before and 4, 8, 12 weeks after balloon injury.

### Procedures

The pigs were anesthetized with ketamine and xylazine intramuscularly, and maintained with isoflurane by ventilator after intubation. Continuous hemodynamic and electrocardiographic monitoring was done. Cutdown was performed on either the external carotid or femoral artery and a 6Fr arterial sheath positioned for vascular access. All animals received a single dose of aspirin (330 mg) two days before procedure. After coronary angiography (CAG) with systemic heparinization (5000 IU/body), left anterior descending and circumflex arteries were dilated three times for 20 seconds using an oversize balloon (Angioscurpt, Volcano Japan; diameter; 3.0–3.5 mm; length,18 mm) at a 1.1–1.2 ratio of balloon to artery diameter.

### Tissue harvesting

Two, 4, 8 and 12 weeks after angioplasty, coronary angiography was repeated as described above. The pigs were euthanized using intravenous barbiturate commercial euthanasia solution and the 46 coronary arteries harvested and perfused with 10% buffered formalin and Coronary artery segments injured by the balloon were paraffin embedded, stained with hematoxylin-eosin, Elastic van Gieson and Azan, and at least two 5-mm long sections of left anterior descending and left circumflex arteries were histomorphometrically analyzed. Subsegments of interest in OCT compound were snap-frozen in liquid nitrogen and cut into 5 μm thick section for oil red. The sections were examined by microscopy and their areas measured using a KS-400 image-analysis system (Carl Zeiss vision GmbH, Hallbermoss, Germany). Injured areas encroached to the external elastic lamina (EEL), the internal elastic lamina (IEL) and lumen areas were measured using computer-assisted digital planimetry. Intimal (plaque) area was calculated using computer-assisted digital planimetry as the IEL area minus lumen area. Observers were blinded to the experimental groups histologically analyzed all sections. Segments of ascending aorta, carotid artery and right coronary artery were also paraffin embedded, stained with hematoxylin-eosin as non-injured vessels.

### Histopathological analysis

Paraffin-embedded slides were deparaffinized, rehydrated and then antigen retrieved by heat induction. A goat polyclonal cathepsin-S antibody (Santa Cruz Biotechnology Inc.) was applied for 30 min at a concentration of 3 μg/mL as described to identify macrophages [16]. Tissue sections were visualized using a NeXES IHC automatic immunostainer (Ventana Medical Systems, Tucson, AZ, USA) and a standard 3’, 3’-diaminobenzidine (DAB) detection kit (Ventana Medical Systems) and counterstained with hematoxylin. The sections were dehydrated, placed in xylene and coverslipped. The ratio (%) of the cathepsin S positive area was determined under high-power magnification (× 400) using a KS-400 image analyzing system (Carl Zeiss Vision GmbH). Anti-alpha smooth muscle actin staining (1:300, Abcam) were also performed.

### Statistical analysis

Experimental values are shown as means and SD unless indicated otherwise. Groups were compared using one-way analysis of variance (ANOVA) or Mann-Whitney U-test. The intima-media ratio in each group was compared using a linear trend test. The significance of differences in positive catepsin-S areas among groups was determined by Tukey’s multiple comparison test. All data were statistically analyzed using JMP8.0 (SAS Institute Inc., Cary, NC, USA). P-value < 0.05 was considered to indicate statistical significance.

## Results

### Body Weight

Table 1 shows time dependent change of body weight and age-matched body weight. Mean body weight at baseline was 602.8 ± 104.4 mg/dL and gradually increased after starting the high-cholesterol/high-fat diet and was maintained in the range of 50–60 kg.

**Table 1:** Time dependent change of body weight (BW).

	baseline	at balloon injury	4week	8week	12week
BW (kg)	29.0±7.9	33.7±3.3	35.2±4.6	56.5±25.8	59.1±32.8
BW/age (kg/day)	0.29±0.06	0.30±0.01	0.24±0.02	0.33±0.11	0.29±0.13

### Serum Lipids

**Fig 2:**
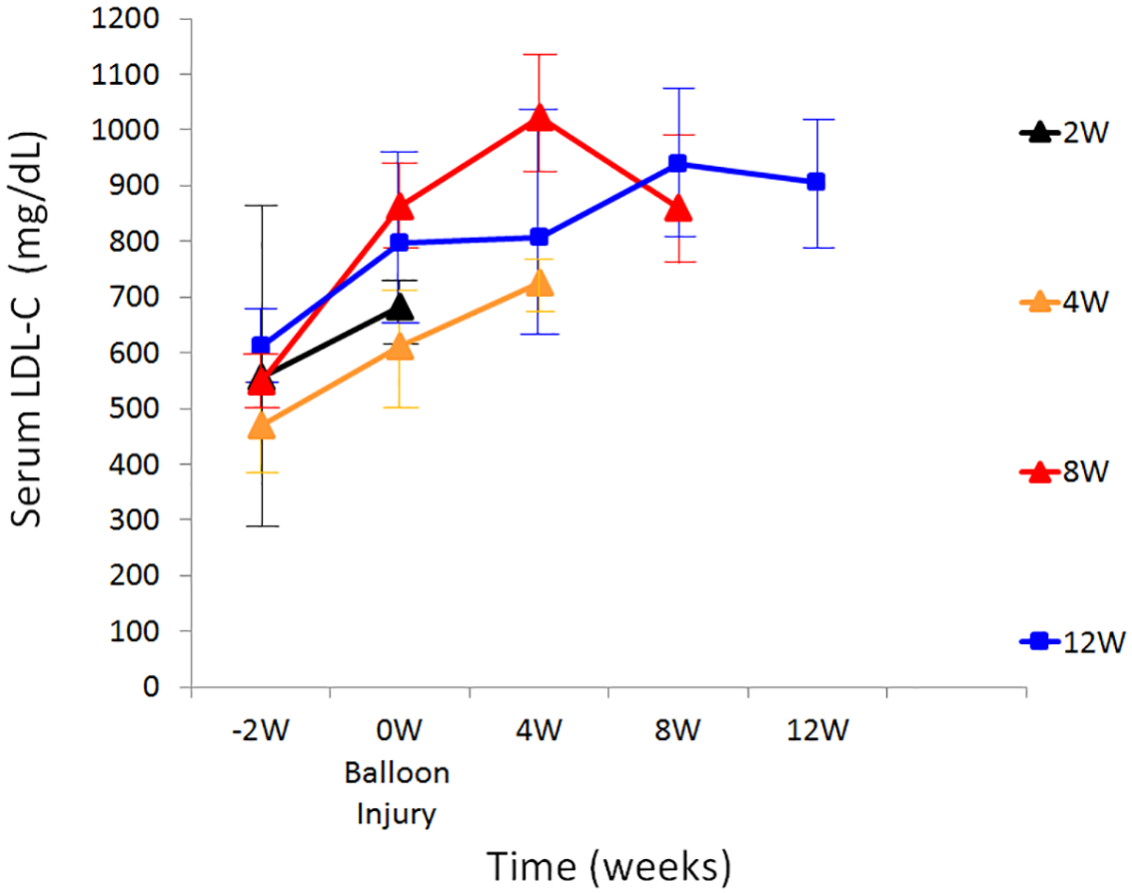
Time dependent changes in low density lipoprotein cholesterol levels in pigs fed with high-cholesterol/high-fat diet (n = 3 each, 12 in total).

Mean baseline levels of total cholesterol and LDL-cholesterol (LDL-C) were 602.8 ± 104.4 mg/dL and 510.0 ± 86.1 mg/dL, respectively (Table 2). Fig 2 shows the serum cholesterol levels of LDLR-KO swine during the study period. LDL-C levels rapidly increased after starting the high-cholesterol/high-fat diet and were maintained in the range of 600 to 1000 mg/dL.

**Table 2:** Lipid Profiles.

Lipid profiles	baseline	4week	8week	12week
TC (mg/dL)	602.3±104.4	904.8±51.7	872.5±46.0	1088.3±294.2
LDL-C (mg/dL)	510.0±86.1	884.8±47.3	859.2±52.4	816.5±61.6
HDL-C (mg/dL)	24.5±7.45	32.6±2.42	34.8±0.71	42.0±1.2
TG (mg/dL)	43.2±10.1	33.8±3.36	42.4±7.6	48.0±28.0

Abbreviations: HDL-C, high density lipoprotein-cholesterol; LDL-C, low density lipoprotein-cholesterol; TC, total cholesterol; TG, triglycelide.

### Non-injured vessels

**Fig 3:**
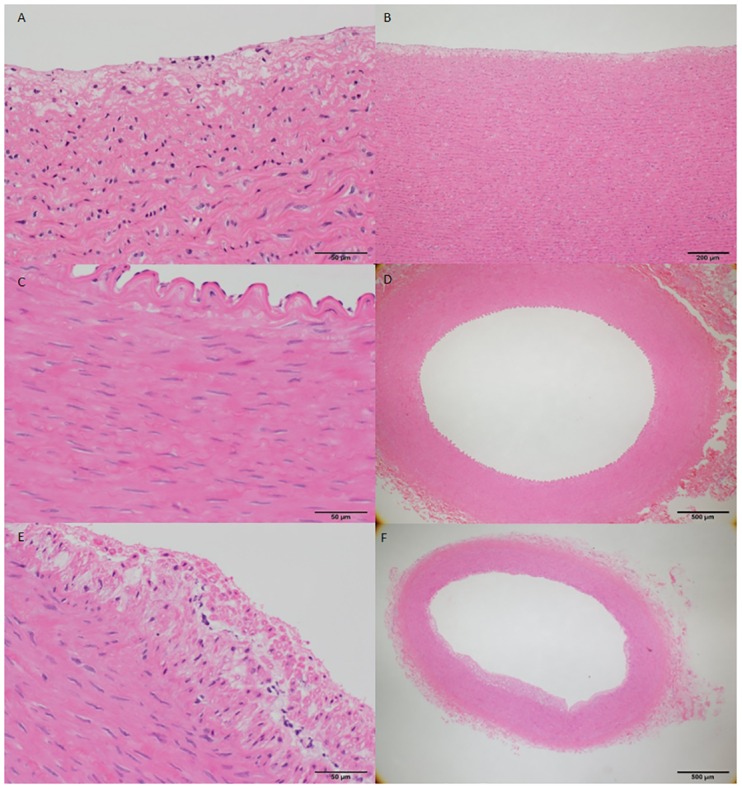
(A-F): Histological features of non-injured vessels of 12 week model. A and B, ascending aorta; C and D, carotid artery; E and F, right coronary artery). Intimal thickening with extracellular lipid accumulation in the luminal part of an arterial intima. Scale bar, 50 μm on the left panel and 200–500 um on the right panel.

Fig 3 shows pathological features of ascending aorta, carotid artery and right coronary artery as non-injured vessels. Intimal thickening with extracellular lipid accumulation in the luminal part of an arterial intima was observed.

### Coronary balloon-injured lesions

**Fig 4:**
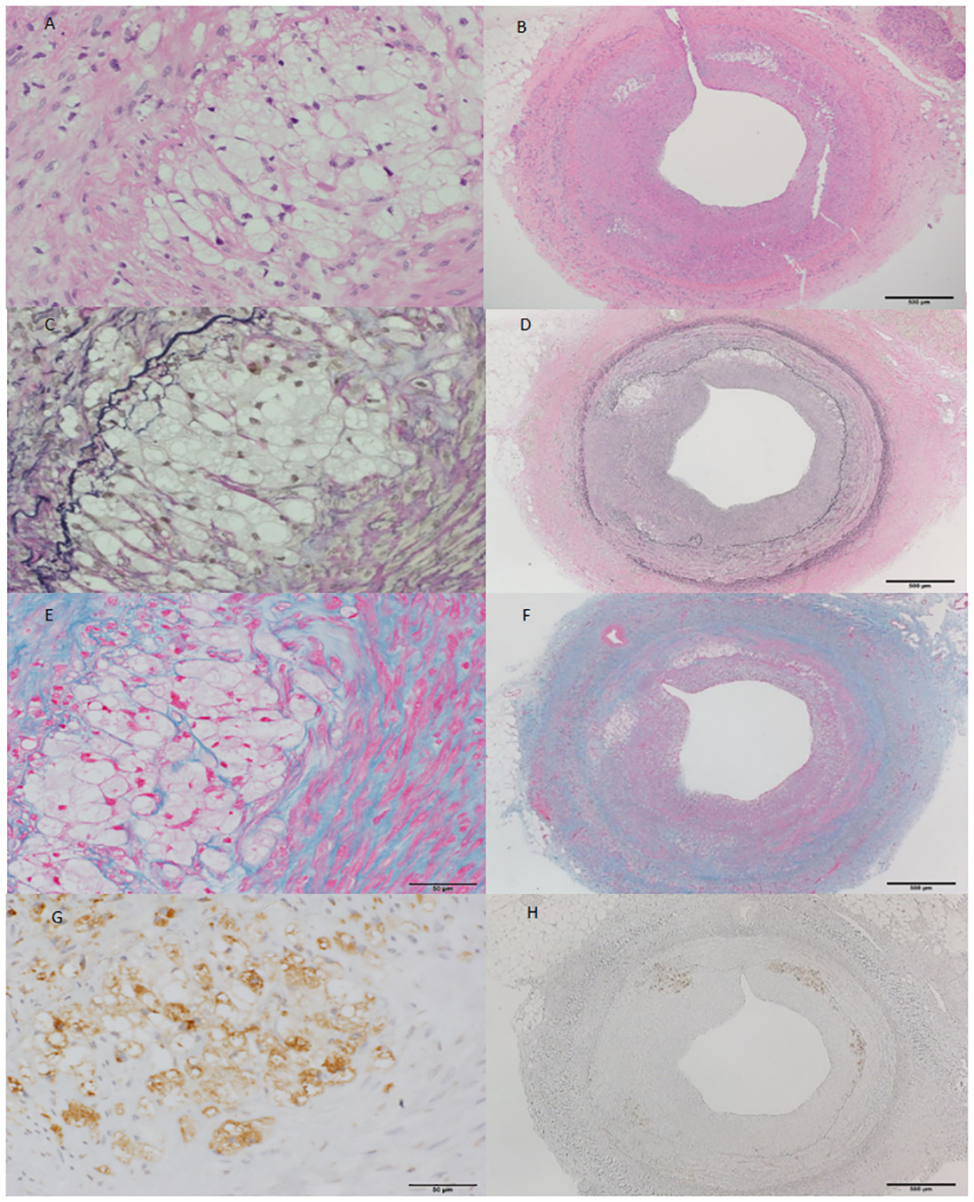
(A-H): Representative coronary section of LDLR-KO pig at four weeks after balloon injury. Intimal hyperplasia consists of accumulated lipid and dense macrophage infiltration. A and B, hematoxylin-eosin; C and D, Elastica van Gieson; D and E, Azan; G and H, cathepsin S immunohistochemical staining. Scale bar, 500 μm on the left panel and 50 μm on the right panel.

**Fig 5:**
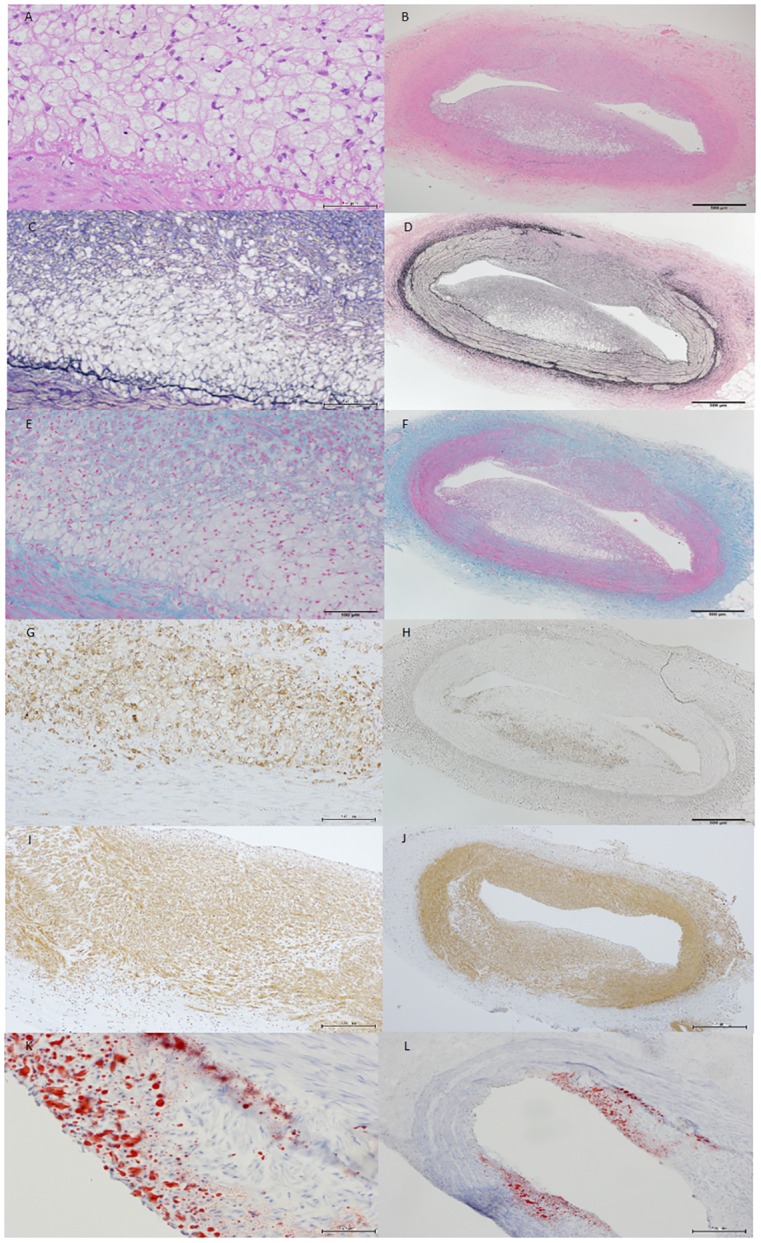
(A-L): Coronary arterial lesion in LDLR-KO pig at eight weeks. Eccentric coronary plaque with considerable macrophage infiltration and foam cells indicates increased lesion complexity. A and B, hematoxylin-eosin; C and D, Elastica van Gieson; E and F, Azan; H and E, cathepsin S immunohistochemical staining; I and J, alpha smooth muscle actin immunohistochemical staining; K and L, oil red staing. Scale bar, 100 μm on the left panel and 500 μm on the right panel.

**Fig 6:**
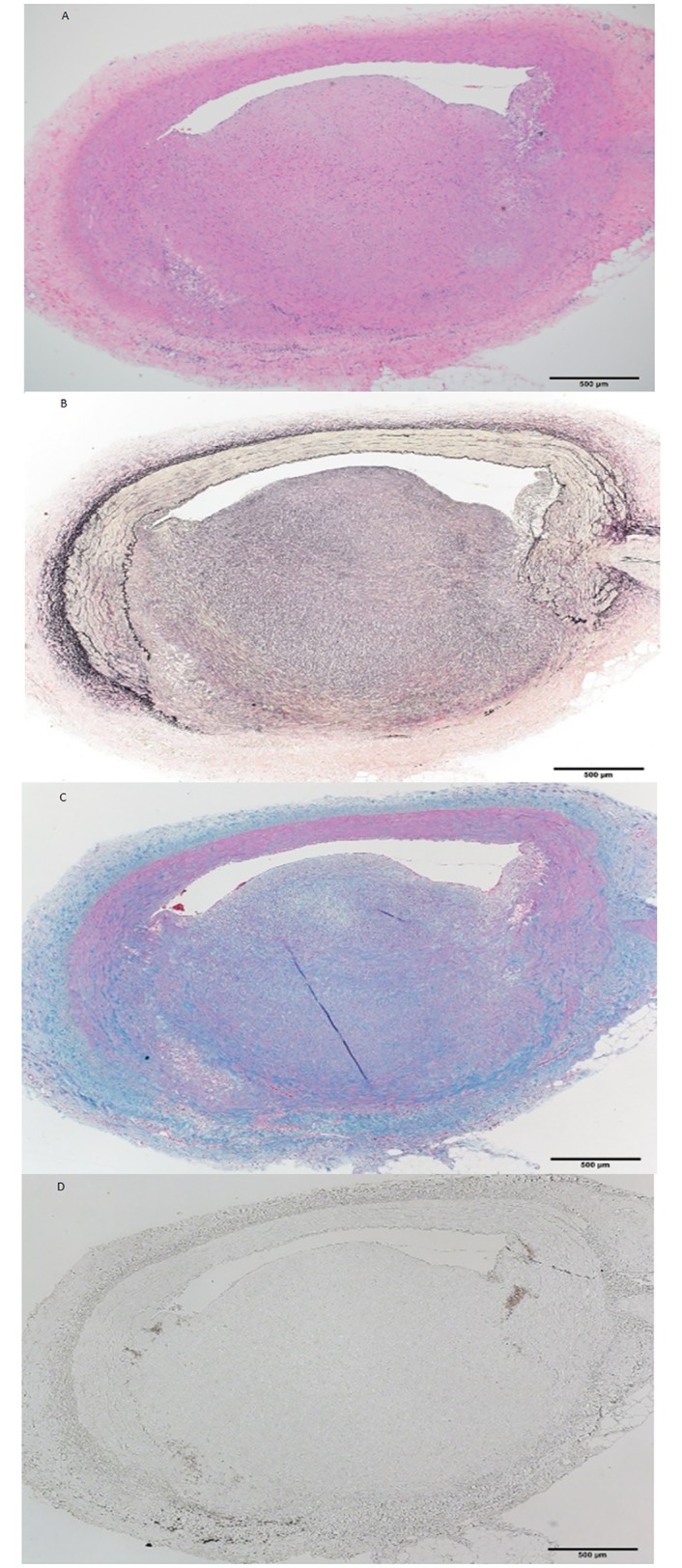
(A-D): Coronary arterial lesion in LDLR-KO pig at 12 weeks. Intima has further thickened and cell components have changed. Numbers of inflammatory cells have decreased and fibrous components have become predominant. A, hematoxylin-eosin; B, Elastica van Gieson; C, Azan; D, cathepsin S immunohistochemical staining. Scale bar, 500 μm.

Table 3 shows pathologic findings. Forty-six coronary artery sections were obtained 2, 4, 8 and 12 weeks after balloon injury (n = 3 each, 12 in total) to evaluate lesion progression. The mean intima to media ratio significantly and dose-dependently increased between weeks 8 and 12 (0.78% ± 0.25% vs. 1.12% ± 0.6%; P for trend, < 0.0001) indicating continuous intimal proliferation even 4 weeks after balloon injury. Coronary lesions at week 4 generally consisted of lipid accumulation with foam cells and inflammatory cells (Fig 4). By week 8, eccentric coronary plaque became infiltrated with macrophages, which together with foam cells indicated increasing lesion complexity (Fig 5). The number of inflammatory cells decreased by week 12 and a fibrous component predominated (Fig 6).

**Table 3:** Pathological values at weeks 2, 4 8 and 12 after balloon-induced arterial injury.

	2 week	4 week	8 week	12 week
Vessel Area (mm^2^)	2.73±0.85	3.10±1.10	3.84±0.53	4.56±1.34
Intima Area (mm^2^)	0.29±0.16	0.79±0.77	1.35±0.40	1.84±0.91
Medial Area (mm^2^)	1.60±0.47	1.60±0.59	1.80±0.39	1.95±0.88
Intima/Media Ratio	0.17±0.06	0.51±0.54	0.78±0.25	1.12±0.66

Variables were represented by mean±SD.

### Time-dependent changes of percent positive cathepsin S area

**Fig 7:**
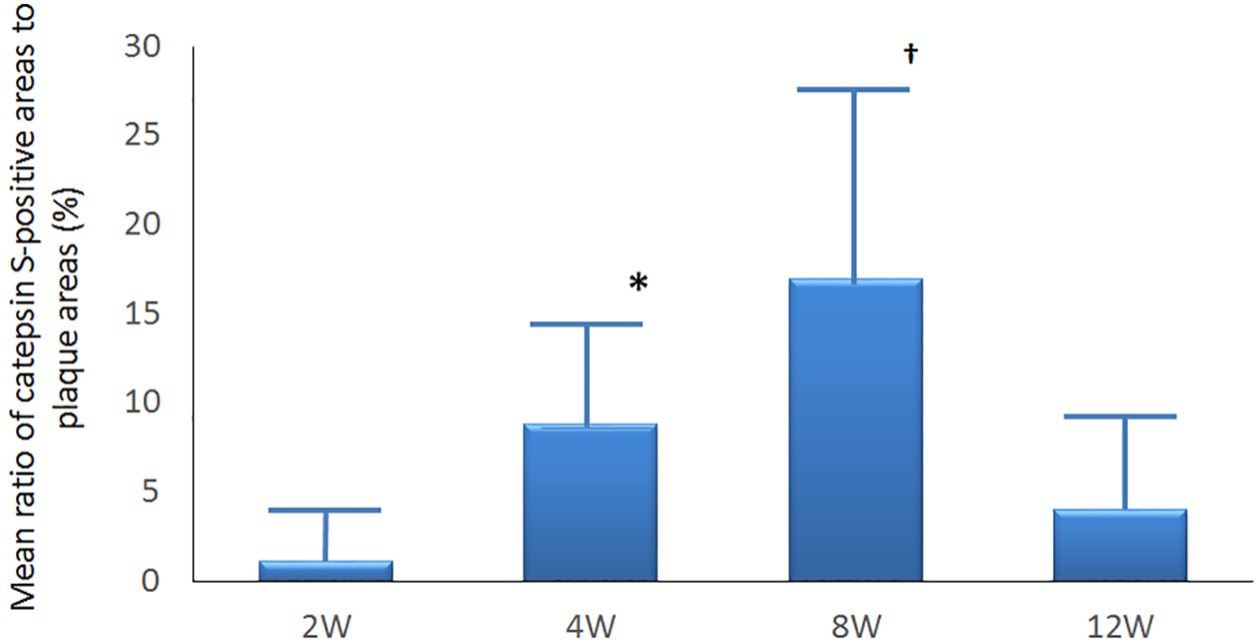
Time dependent changes in ratio of area positively stained for cathepsin S. Macrophages in lesions were immunohistochemically stained with cathepsin S (n = 12). Mean ratios of macrophages to plaque areas significantly and time-dependently changed at weeks 4* and 8^†^ compared with that at week 2 (8.79% ± 5.98% and 17.00% ± 10.38% vs. 1.14% ± 1.88%; P < 0.0001) whereas at week 12 the ratio decreased towards the value at week 2 (4.00% ± 4.56% vs; baseline P = 0.66).

Macrophage content in lesions was immunohistochemically quantified using cathepsin S. The mean ratios of macrophages to plaque areas were significantly and time dependently higher at weeks 4 and 8 compared with week 2 (8.79% ± 5.98% and 17.00% ± 10.38% vs 1.14% ± 1.88%; P < 0.0001 for both), whereas the ratio (%) of the cathepsin S area had decreased to 4.00% ± 4.56% (P = 0.66 vs. baseline) at week 12 (Fig 7).

## Discussion

We developed a large animal coronary atherosclerosis model for evaluating coronary atherosclerosis. This was accomplished using a LDLR-KO pig created by deleting the LDLR exon region from cultured porcine fetal fibroblasts. Mean baseline LDL-C levels were higher than those of other swine with familial hyperlipidemia. Moreover, coronary atherosclerosis was rapidly and reproducibly created by feeding the LDLR-KO pig with a high-cholesterol/high-fat diet and injuring their coronary arteries using balloons. We also discovered a significant time-dependent variation of histological lesion characteristics of coronary arterial lesions

A large animal model of coronary atherosclerosis similar to that in humans with respect to the size of coronary arteries and lesion characteristics is essential for translational studies in coronary intervention and imaging. Other pig models of advanced coronary atherosclerosis are less appropriate for such studies since pathology is quite variable and the long period required. Diet-induced mild-to-moderate atherosclerotic lesions have created in swine [8, 9] and Rapacz introduced familial hyperlipidemic pigs with advanced coronary atherosclerotic lesions[17, 18]. However, they have not been used in preclinical studies because up to two years is required for advanced lesions, the weight of pigs becomes unwieldy (> 200 kg) and they are extremely expensive to produce and maintain. Several line of gene-modified pigs for atherosclerosis have also been reported [19–22]. David et al generated Yucatan miniature pigs with targeted disruptions of the LDLR gene showing advanced coronary atherosclerotic lesions at 11 months of age. Al-Mashhadi et al also generated Yucatan minipigs of atherosclerosis model induced by DNA transposition of a PCSK9 gain-of function mutations. These model using miniature pig takes advantages in size and consistency over previous models. Recently Li Y et al and our co-worker (Onishi A) generated a cloned LDL-R-targeted pig and showed spontaneous coronary atherosclerosis with high fat diet at 7 months of age [13]. We also confirmed early stage of spontaneous atherosclerosis in ascending aorta, carotid artery and right coronary artery as non-injured vessels using the same line of LDLR KO pig as our model in 12 week model (at 5 months of age) but not in earlier model.

General limitations to porcine coronary atherosclerotic models include the need for significant infrastructure to support animal maintenance, a long time frame to create and monitor lesions and significant financial investment. Significant coronary atherosclerotic lesions take a long time to develop and they do so in unpredictable locations in current models of naturally-occurring atherosclerosis. A more manageable and reproducible model has been developed by accelerating the atherosclerotic process in susceptible animals. Granada et al developed a novel coronary atherosclerotic model using percutaneous intramural injections of cholesteryl linoleate. This model expressed complex, inflammatory lesions that contained lipid and resembled those of complex human atherosclerotic plaque on intravascular ultrasound images [23]. However, the major limitation of this model is that lesion contain an abundant smooth muscle cells/proteoglycans but without a necrotic core, calcification, and collagen type I [24]. Thim T et al. recently developed a downsized pig with familial hypercholesterolemia and created human-like coronary atherosclerosis by balloon-injuring the coronary artery with balloons after 14 weeks suggesting that the high-cholesterol/high-fat diet together with balloon injury accelerate coronary atherosclerosis [25]. Exposing injured arteries to extremely high levels of LDL-C might have been responsible for accelerating the formation of coronary atherosclerotic lesions in our model. In our model, we showed advanced coronary atherosclerotic lesion at an earlier period (8 weeks after balloon injury) and less expensive than downsized pigs which may take advantages in time frame for animal management and financial investment.

Coronary angioplasty is an established procedure in domestic swine with normal cholesterol levels. The key problem with this model is that the arteriosclerotic pathology differs from that in human; the lesions have abundant fibrotic tissue with little necrotic core formation [26]. Our investigation of temporal changes in histological tissues from our model pigs revealed human-like atherosclerotic lesions containing a lipid pool at four and eight weeks, and predominant fibrous components at 12 weeks. The serial time course of atherosclerosis process appeared to be accelerated in our model.

## Limitations

The present study has limitations. We combined diet-induced hypercholesterolemia with balloon injury to accelerate the atherosclerotic process and reduce the observation period in a small sample of LDLR-KO pigs. Human atherosclerosis naturally develops without local lesion injury and local accelerated model lesions might not be representative of spontaneous coronary lesions. Compared with the natural course of human atherosclerosis, our model produced plaque containing smooth muscle cells derived from vascular injury. However, obstructive atherosclerosis in humans is clinically treated using non-compliant balloons and stents, therefore, injury induced in a model might also be helpful to verify the effects of such treatment modalities [27]. Although spontaneous atherosclerotic lesions might differ, accelerated lesions can be useful in studies of specific atherosclerosis-related processes [28, 29] and in imaging studies that detect a specific localized plaque component without the unpredictability of locations associated with spontaneous lesions [7].

## Conclusions

We established a model of accelerated coronary atherosclerosis using LDLR-KO pigs with balloon injury. This model can be useful as a preclinical tool for pharmacology or devices and as an aid to investigate the mechanism of plaque formation and progression.

## Abbreviations

LDL-C: low density lipoprotein cholesterol
LDLR: low density lipoprotein receptor
LDLR-KO: low density lipoprotein receptor knock-out
TG: triglyceride

## References

[1] Roger VL, Go AS, Lloyd-Jones D, Adams RJ, Berry JD, Brown TM, et al; American Heart Association Statistics Committee and Stroke Statistics Subcommittee. Heart disease and stroke statistics-2011 update: a report from the American Heart Association. *Circulation* 2011; 123:e18–e209. doi: 10.1161/CIR.0b013e3182009701 21160056 10.1161/CIR.0b013e3182009701 PMC4418670

[2] Rosenfeld ME, Polinsky P, Virmani R, Kauser K, Rubanyi G, Schwartz SM. Advanced atherosclerotic lesions in the innominate artery of the ApoE knockout mouse. *Arterioscler Thromb Vasc Biol* 2000;20:2587–2592. 11116057 10.1161/01.atv.20.12.2587

[3] Shiomi M, Ito T. The Watanabe heritable hyperlipidemic rabbit, its characteristics and history development: A tribute to the late Dr. Yoshio Watanabe. *Atherosclerosis* 2009;207:1–7. doi: 10.1016/j.atherosclerosis.2009.03.024 19389675 10.1016/j.atherosclerosis.2009.03.024

[4] Hasler-Rapacz J, Ellegren H, Fridolfsson AK, Kirkpatrick B, Kirk S, Andersson L, et al Identification of a mutation in the low density lipoprotein receptor gene associated with recessive familial hypercholesterolemia in swine. *Am J Med Genet* 1998;76:379–386. 9556295

[5] Prescott MF, Hasler-Rapacz J, Von Linden-Reed J, Rapacz J. Familial hypercholesterolemia associated with coronary atherosclerosis in swine bearing different alleles for apolipoprotein B. *Ann N Y Acad Sci* 1995;748:283–292. 7695172 10.1111/j.1749-6632.1994.tb17326.x

[6] Wernersson R, Schierup MH, Jorgensen FG, Gorodkin J, Panitz F, Staerfeldt HH, et al Pigs in sequence space.: a 0.66X coverage pig genome survey based on shotgun sequencing. BMC Genomics 2005;6:70 15885146 10.1186/1471-2164-6-70 PMC1142312

[7] Granada JF, Kaluza GL, Wilensky RL, Biedermann BC, Schwartz RS, Falk E. Porcine models of coronary atherosclerosis and vulnerable plaque for imaging and interventional research. Eurointervention 2009;5:140–148. 19577996 10.4244/eijv5i1a22

[8] Suzuki Y, Yeung AC, Ikeno F. The pre-clinical animal model in the translational research of interventional cardiology. *J Am Coll Cardiol Interv* 2009;2:373–383. 10.1016/j.jcin.2009.03.004 19463458

[9] Reitman JS, Mahley RW, Fry DL. Yucatan miniature swine as a model for diet-induced atherosclerosis. *Atherosclerosis* 1982;43:119–132 7092978 10.1016/0021-9150(82)90104-6

[10] Thorpe PE, Hunter WJ, Zhan XX, Dovgan PS, Agrawal DK. A noninjury, diet-induced swine model of atherosclerosis for cardiovascular-intervention research. *Angiology* 1996;47:849–857. 8810651 10.1177/000331979604700903

[11] Gerrity RG, Natarajan R, Nadler JL, Kimsey T. diabetes-induced accelerated atherosclerosis in swine. *Diabetes* 2001;50:1654–1665. 11423488 10.2337/diabetes.50.7.1654

[12] Granada JF, Moreno PR, Burke AP, Schulz DG, Raizner AE, Kaluza GL. Endovascular needle injection of cholesteryl linoleate into the arterial wall produces complex vascular lesions identifiable by intravascular ultrasound: early development in a porcine model of vulnerable plaque. *Coron Artery Dis* 2005;16:217–224. 15915073 10.1097/00019501-200506000-00002

[13] Li Y, Fuchimoto D, Sudo M, Haruta H, Lin QF, Takayama T, et al Development of human-like advanced coronary plaques in low-density lipoprotein receptor knockout pigs and justification for statin treatment before formation of atherosclerotic plaques. J Am Heart Asssoc 2016;5pii:e002779. 10.1161/JAHA.115.002779 PMC4843535 27091180

[14] Onishi A, Iwamoto M, Akita T, Mikawa S, Takeda K, Awata T, et al Pig cloning by microinjection of fetal fibroblast nuclei. Science 2000;289:1188–1190. 10947985 10.1126/science.289.5482.1188

[15] Kikuchi K, Onishi A, Kashiwazaki N, Iwamoto M, Noguchi J, Kaneko H, et al Successful piglet production after transfer of blastocysts produced by a modified in vitro system. Biol Reprod 2002;66:1033–1041. 11906923 10.1095/biolreprod66.4.1033

[16] Wilensky RL, Shi Y, Mohler ER III, Hamamdzic D, Burgert ME, Li J, et al Inhibition of lipoprotein-associated phospholipase A2 reduces complex coronary atherosclerotic plaque development. *Nat Med* 2008;14:1059–1066. doi: 10.1038/nm.1870 18806801 10.1038/nm.1870 PMC2885134

[17] Rapacz J, Hasler-Rapacz J, Taylor KM, Checovich WJ, Attie AD. Lipoprotein mutations in pigs are associated with elevated plasma cholesterol and atherosclerosis. *Science* 1986;234:1573–1577. 3787263 10.1126/science.3787263

[18] Prescott MF, McBride CH, Hasler-Rapacz J, Von LJ, Rapacz J. Development of complex atherosclerotic lesions in pigs with inherited hyper-LDL cholesterolemia bearing mutant alleles for apolipoprotein B. *Am J Pathol* 1991;139:139–147. 1853929 PMC1886122

[19] Davis BT, Wang XJ, Rohret JA, Struzynski JT, Merricks EP, Bellinger DA, et al Targeted disruption of LDLR causes hypercholesterolemia and atherosclerosis in Yucatan miniature pigs. PLoS One 2014;9:e93457 doi: 10.1371/journal.pone.0093457 24691380 10.1371/journal.pone.0093457 PMC3972179

[20] Ozawa M, Himaki T, Ookutsu S, Mizobe Y, Ogawa J, Miyoshi K, et al Production of cloned miniature pigs expressing high levels of apolipoprotein (a) in plasma. PLoS One 2015;10:e0132155 doi: 10.1371/journal.pone.0132155 26147378 10.1371/journal.pone.0132155 PMC4492603

[21] Al-Mashhadi RH, Sorensen CB, Kragh PM, Chirstoffersen C, Mortensen MB, Tolbod LP, et al Familial hypercholesterolemia and atherosclerosis in cloned minipigs created by DNA transposition of a human PCSK9 gain-of function mutant. Sci Transl Med 2013;5:166ra1 doi: 10.1126/scitranslmed.3004853 23283366 10.1126/scitranslmed.3004853

[22] Jensen TW, Mazur MJ, Pettiew JE, Perez-Mendoza VG, Zachary J, Schook LB. A cloned pig model for examining atherosclerosis induced by high fat, high cholesterol diets. Anim Biotechnol 2010;21:179–187. doi: 10.1080/10495398.2010.490693 20665290 10.1080/10495398.2010.490693

[23] Gradada JF, Wallace-Bradley D, Win HK, Alviar CL, Builes A, Lev EI, et al In vivo plaque characterization using intravascular ultrasound-virtual histology in a porcine model of complex coronary lesions. *Arterioscler Thromb Vasc Biol* 2007;27:387–393. 17138936 10.1161/01.ATV.0000253907.51681.0e

[24] Virmani R, Nakazawa G. Animal models and virtual histology. *Arterioscler Thromb Vasc Biol* 2007;27:1666, author reply 1667–1668. 17581831 10.1161/ATVBAHA.107.143198

[25] Thim T, Hagensen MK, Drouet L, Bal Dit Sollier C, Bonneau M, Granada JF, et al Familial hypercholesterolemic downsized pig with human-like coronary atherosclerosis: a model for preclinical studies. *EuroIntervention* 2010;6:261–268. doi: 10.4244/ 20562079 10.4244/EIJV6I2A42

[26] Maeng M, Olesen PG, Emmertsen NC, Thorewest M, Nielsen TT, Kristensen BO, et al Time course of vascular remodeling, formation of neointima, and formation of neoadventitia after angioplasty in a porcine model. *Coron Artery Dis* 2001;12:285–293. 11428537 10.1097/00019501-200106000-00004

[27] Thim T. Human-like atherosclerosis in minipigs: a new model for detection and treatment of vulnerable plaques. *Dan Med Bull* 2010;57:B4161 20591344

[28] Bentzon JF, Weile C, Sondergaard CS, Hindkjaer J, Kassem M, Falk E. Smooth muscle cells in atherosclerosis originate from the local vessel wall and not circulating progenitor cells in Apo E knockout mice. Arterioscler Thromb Vasc Biol 2006;26:2696–2702. 17008593 10.1161/01.ATV.0000247243.48542.9d

[29] Bentzon JF, Sondergaard CS, Kassem M, Falk E. Smooth muscle cells healing atherosclerotic plaque disruptions are of local, not blood, origin in apolipoprotein E knockout mice. Circulation 2007;116:2053–2061. 17938286 10.1161/CIRCULATIONAHA.107.722355

